# Evolution of Postpartum Weight and Body Composition after Excessive Gestational Weight Gain: The Role of Lifestyle Behaviors—Data from the INTER-ACT Control Group

**DOI:** 10.3390/ijerph18126344

**Published:** 2021-06-11

**Authors:** Margriet Bijlholt, Lieveke Ameye, Hanne van Uytsel, Roland Devlieger, Annick Bogaerts

**Affiliations:** 1 Centre for Research and Innovation in Care (CRIC), Faculty of Medicine and Health Sciences, University of Antwerp, 2610 Antwerp, Belgium; margriet.bijlholt@uantwerpen.be; 2 Unit Woman and Child, KU Leuven, Department of Development and Regeneration, 3000 Leuven, Belgium; lieveke.ameye@kuleuven.be (L.A.); hanne.vanuytsel@kuleuven.be (H.v.U.); roland.devlieger@uzleuven.be (R.D.); 3 Department of Obstetrics and Gynecology, University Hospital Leuven, 3000 Leuven, Belgium; 4 Faculty of Health, University of Plymouth, Devon PL4 8AA, UK

**Keywords:** eating behavior, emotional eating, sedentary behavior, uncontrolled eating, maternal health, obesity, overweight, postpartum weight retention

## Abstract

Women with excessive gestational weight gain are at increased risk of postpartum weight retention and potentially also unfavorable body composition. Insight into the lifestyle behaviors that play a role in the evolution of postpartum weight and body composition among these women could aid identification of those at highest risk of long-term adverse outcomes. This secondary analysis of the INTER-ACT randomized controlled trial investigates control group data only (n = 524). The evolution of weight retention, percentage loss of gestational weight gain, fat percentage, waist circumference, and associated lifestyle behaviors between 6 weeks and 12 months postpartum were assessed using mixed model analyses. At six weeks postpartum, every sedentary hour was associated with 0.1% higher fat percentage (*P* = 0.01), and a higher emotional eating score was associated with 0.2% higher fat percentage (*P* < 0.001) and 0.3 cm higher waist circumference (*P* < 0.001). Increase in emotional eating score between 6 weeks and 6 months postpartum was associated with a 0.4 kg (*P* = 0.003) increase in postpartum weight retention from six months onwards. Among women with overweight, an increase in the uncontrolled eating score between 6 weeks and 6 months postpartum was associated with a 0.3 kg higher postpartum weight retention (*P* = 0.04), and 0.3% higher fat percentage (*P* = 0.006) from six months onwards. In conclusion, sedentary and eating behaviors play important roles in postpartum weight and body composition of women with excessive gestational weight gain and should therefore be incorporated as focal points in lifestyle interventions for this population.

## 1. Introduction

The retention of pregnancy weight after delivery contributes to long-term overweight and associated morbidities such as cardiovascular diseases, type 2 diabetes mellitus and certain cancer types [1,2,3,4]. Postpartum weight normalization is therefore highly recommended. However, 75% of women do not return to their pre-pregnancy weight in the year following delivery [1]. Studies show a mean postpartum weight retention (PPWR) of four to five kilograms one year after delivery [1,5]. An important risk factor for PPWR is excessive gestational weight gain (GWG) [5,6], which occurs in approximately 35% to 50% of pregnancies [7,8]. Women with an excessive GWG were previously found to have a three kilogram higher PPWR three years after delivery compared to women who had an adequate GWG [9]. It is therefore of great public health importance that women with previous excessive GWG receive special attention for the reduction of PPWR.

Aside from GWG, maternal age, race, employment status, breastfeeding, physical activity and food intake are suggested to affect PPWR [10,11,12,13,14]. Certain eating behaviors might also play a role, as a recent systematic review shows that more restrained and intuitive eating are potentially related to decreased postpartum weight retention [15]. Other eating behaviors such as emotional eating and uncontrolled eating have not yet been studied in relation to PPWR, though they are associated with weight in the general population [16,17]. It should be investigated whether these determinants play a role in PPWR and specifically among women with excessive GWG.

Weight and body mass index (BMI) are commonly reported measures in postpartum studies, yet these measures alone do not provide a complete picture of body composition. To illustrate, BMI is correlated with fat mass, but fat percentage can strongly differ between individuals with the same BMI [18,19]. An increased fat percentage is associated with metabolic syndrome and cardiovascular risk [20,21]. BMI cannot establish central adiposity, which is likewise related to metabolic syndrome and cardiovascular diseases [22,23]. Waist circumference is a useful and accurate measure of central adiposity [18,24]. Taking into account fat percentage and waist circumference in addition to weight and BMI is therefore of added value.

Although body composition is related to lifestyle factors such as food intake and physical activity among general populations [25,26], little research is available that investigates relationships between lifestyle and body composition in the postpartum period. In addition, more knowledge on the evolution and factors associated with PPWR specifically among women with excessive GWG could aid in the development of interventions targeting this high-risk group, as targeted interventions are currently unavailable [27]. Therefore, the aim of this paper is to investigate whether lifestyle factors including food intake, eating behavior, physical activity and sedentary behavior as well as breastfeeding are associated with PPWR, percentage GWG loss (% GWGL), fat percentage and waist circumference changes in the first year after delivery among women with excessive GWG.

## 2. Materials and Methods

The present study is a secondary analysis of the INTER-ACT randomized controlled trial (RCT). For the purpose of the current analyses, data from control group participants of the INTER-ACT RCT were used. The methodology of the INTER-ACT study is described in detail elsewhere [28]. In brief, the INTER-ACT RCT provided a blended care lifestyle intervention to women with preceding excessive GWG, with the aim to reduce pregnancy- and birth-related complications in a next pregnancy. The intervention and data collection comprised two phases: a postpartum phase and a subsequent pregnancy phase. Postpartum data collection took place at six weeks, six months and twelve months after delivery. Subsequently, data was collected every six months until a next pregnancy. Pregnancy data collection took place once in every trimester of the pregnancy. For the purpose of the current analyses, only the postpartum data collection at six weeks, six months and twelve months after delivery for the control group participants was included. Outcomes of interest in the current analyses were postpartum weight retention, percentage of GWG lost, fat percentage and waist circumference. More details on these outcomes are described in Section 2.6 on anthropometry.

### 2.1. Recruitment

From May 2017 until April 2019, participants were recruited 2 to 3 days after delivery by research midwives in six hospitals in three different regions in Flanders, Belgium: University Hospital Leuven, University Hospital Antwerp, Gasthuiszusters Hospital Antwerp, St-Franciscus Hospital in Heusden-Zolder, Jessa Hospital in Hasselt and Hospital Oost-Limburg in Genk.

### 2.2. Eligibility Criteria

Women were eligible for participation in the study if they had excessive GWG in the preceding pregnancy according to the National Academy of Medicine guidelines, being a GWG > 16 kg for women with normal weight, >11.5 kg for women with overweight and >9 kg for women with obesity [29]. Further eligibility criteria were age ≥ 18 years and being sufficiently proficient in Dutch to independently fill out questionnaires. Women were ineligible for participation after stillbirth, if they delivered twins, if they had a history of bariatric surgery or a planned one, or if they had a chronic disorder such as diabetes mellitus, renal disease or a psychiatric disorder.

### 2.3. Participants

**Figure 1:**
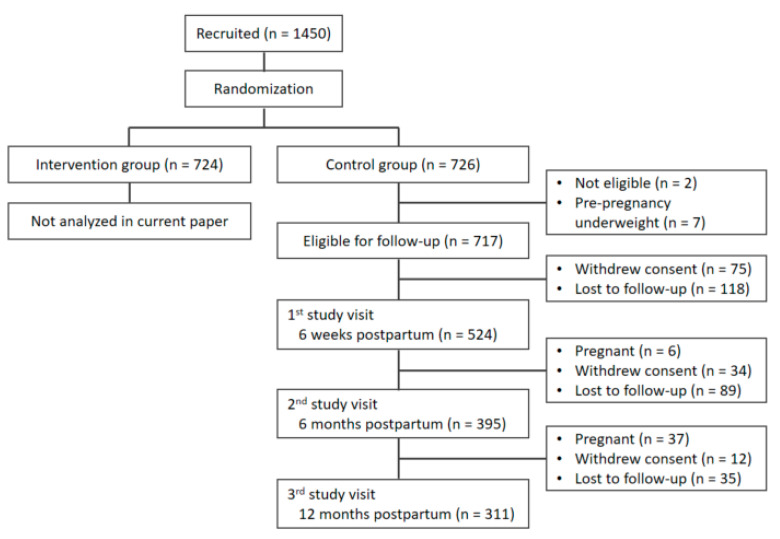
Flow chart of participant follow-up.

Details on the sample size calculation of the INTER-ACT study are described in the study protocol [28]. In the larger RCT, 1450 participants were recruited, of which 724 were randomized into the intervention group and 726 into the control group. Randomization was performed by the electronic Case Report Form (eCRF) system. Blinding could not be applied due to the nature of the RCT. For the current analyses, only the control group participants were selected. They received usual care only. Two participants were removed because they did not meet eligibility criteria. Seven participants had an underweight pre-pregnancy BMI and were excluded from the analyses. This resulted in 717 participants who were eligible for the current analyses. The first study visit (six weeks postpartum) was completed by 524 control group participants; 386 completed the second study visit (six months postpartum); 311 completed the third study visit (12 months postpartum) (Figure 1).

### 2.4. Data Collection

An eCRF was used to capture all data. Data from the medical records were collected at recruitment. Anthropometric and questionnaire data were collected at 6 weeks (median 7.1, IQR 6.3–8.1), 6 months (median 6.1, IQR 5.9–6.4) and 12 months postpartum (median 12.1, IQR 11.9–12.3). A timeframe of two weeks before to four weeks after 6 weeks, 6 months and 12 months postpartum was allowed for the measurements for feasibility. Trained researchers and research assistants collected data at the home of the participant, at the hospital or at a different location in accordance with the preference of the participants. If participants became pregnant again within the first 12 months postpartum, data collected during that pregnancy were excluded from the current analyses.

### 2.5. Medical Record Data

Data on method of conception, parity, sex of the infant and (pre-)pregnancy weight were retrieved from the medical records. Pre-pregnancy weight and weight at delivery were self-reported or measured by a caregiver and were used to calculate GWG and determine excessive GWG according to the National Academy of Medicine guidelines [29].

### 2.6. Anthropometry

Weight and fat mass were determined using a Tanita MC 780 SMA bio-electric impedance device (Tanita Corporation, Tokyo, Japan) with three frequencies (5, 50 and 250 kHz). The measurement was taken in light clothing and bare feet while standing on the scale and holding grips with integrated electrodes for the measurement of body composition. Height was measured with a Seca 213 stadiometer to the nearest 0.1 cm while standing straight with the head oriented in the Frankfurt plane position. Waist circumference was measured to the nearest 0.1 cm midway between the lowest rib and the hip bone using a Seca 201 measuring tape.

Postpartum weight retention is the difference between weight at a given point of time in the postpartum period and pre-pregnancy weight. Percentage gestational weight gain loss (*% GWGL*) was computed for every respective measurement time point (six weeks, six months and twelve months postpartum), as follows:

(1) $$\%\;GWGL=\frac{\mathrm{weight}\;\mathrm{at}\;\mathrm{delivery}\;-\;\mathrm{postpartum}\;\mathrm{weight}}{\mathrm{gestational}\;\mathrm{weight}\;\mathrm{gain}}\times 100\%$$

### 2.7. Questionnaires

Personalized links to the online questionnaires were sent out from the eCRF system. All participants received questionnaires according to a fixed time scheme: two weeks in advance of the appointment; in the case of non-response, reminders were sent one week and two days in advance of the appointment.

Questions on sociodemographic factors such as level of education, employment status and ethnicity were only included in the first questionnaire. Breastfeeding questions were based on previous studies and included items on exclusive breastfeeding, combined or formula feeding and duration of breastfeeding [30]. Depressive feelings in the past were queried in one question based on earlier publications [31,32].

Information on food intake was obtained using a validated 24-item Food Frequency Questionnaire [33]. For every item, frequency and quantity of intake were queried. Total energy and macronutrients were calculated.

Eating behavior was assessed using the validated Three-Factor Eating Questionnaire Revised 18-item version (TFEQ-R18) [34]. Eighteen Likert-scale items queried restrained eating, emotional eating, and uncontrolled eating. Restrained eating refers to “a tendency to constantly and consciously restrict one’s food intake instead of using physiological cues, hunger and satiety, as regulators of food intake”; uncontrolled eating refers to “a tendency to overeat, with the feeling of being out of control”; emotional eating refers to “the tendency to eat in response to negative emotions”[35]. Total scores for every type of eating behavior were converted to a scale ranging from 0 to 100, representing the relative proportion of the highest possible raw scores [35].

The International Physical Activity Questionnaire (IPAQ) was used to assess physical activity and sedentary behavior. The IPAQ includes questions on physical activity related to one’s job, transportation, housework, house maintenance, caring for family, recreation, sport, leisure time and time spent sitting. Physical activity was expressed as the metabolic equivalent of task minutes (MET-minutes) per week, and sedentary time was expressed in minutes per week [36].

### 2.8. Ethical Approval and Informed Consent

Ethical approval for this study was obtained on 9 March 2017 by the medical ethics committees of all six participating hospitals (protocol code B322201730956), and all participants provided informed consent at recruitment. The study was pre-registered on ClinicalTrials.gov (NCT02989142).

### 2.9. Data Analysis

Data were analyzed using SPSS version 27 and SAS version 9.4. Descriptive characteristics were presented as mean and standard deviation or median and interquartile range for continuous variables and number and percentage for categorical variables. Differences in characteristics between dropouts and participants who completed the third study visit were determined using likelihood ratio chi-square test or Fisher Exact test for categorical variables and unpaired t-test or Mann-Whitney U test for continuous variables.

For univariate analyses, the study population was divided into women who had a PPWR < 5 kg and ≥5 kg. This cut-off represents substantial PPWR with potential long-term consequences and has been regularly utilized in previous studies [13,14,37,38]. Differences in categorical variables were assessed with likelihood ratio chi-square test or Fisher Exact test and differences in continuous variables were assessed with unpaired t-test or Mann-Whitney U test. A *P*-value < 0.05 was considered statistically significant. The same tests were performed to compare dropouts to non-dropouts.

For each of the four outcome variables—PPWR, % GWGL, fat percentage and waist circumference evolution—a mixed model was constructed by considering time (in months), pre-pregnancy BMI class, level of education, employment status, method of conception, parity, sex of infant, depressive feelings in the past, amount of excessive GWG, breastfeeding, physical activity, sedentary time, caloric intake, macronutrient intake and eating behavior as possible explanatory variables. For each of the explanatory variables, the interaction term with BMI class was considered (e.g., different effect of breastfeeding on the outcome in obese/overweight compared to normal weight) as was the interaction term with time (stronger/lower effect in obese or overweight, over time). A broken line regression was considered for the time effect, with a break point at 6 months, allowing a difference in effect during the first months postpartum compared to after 6 months postpartum. The mixed models use a random intercept, random slope and unstructured working correlation matrix in order to take into account the dependency between the consecutive body composition measurements of the same mother over time.

## 3. Results

### 3.1. Participant Characteristics

A total of 717 participants were eligible for the current analyses, of which 524 completed the first study visit at six weeks postpartum, 386 the second study visit at six months postpartum, and 311 the third study visit at 12 months postpartum (Figure 1).

Table 1 presents characteristics of the 524 participants who completed at least one study visit. Women who dropped out before the third study visit at 12 months postpartum significantly differed from those who completed the third study visit in terms of education (respectively, 25.1% and 36.2% held a master’s degree, *P* = 0.003), pre-pregnancy BMI (respectively, 40.4% and 54.7% had a normal BMI, *P* = 0.006), GWG among the overweight subgroup (respectively, 17 and 15 kg, *P* = 0.007), breastfeeding (respectively, 49.8% and 59.8% exclusively breastfed at six weeks postpartum, *P* = 0.03), and history of depressive feelings (respectively, 21.1% and 12.3%, *P* = 0.009) (Table S1).

At 12 months postpartum, 30% of the women had returned to their pre-pregnancy weight. Mean % GWGL at 12 months postpartum was 89.2% (SD 32.2). Almost one in four women (23.8%) retained 5 kg or more at 12 months postpartum. Women with ≥5 kg PPWR more often had education up to 18 years of age and less often a master’s degree or higher compared to women with <5 kg PPWR at 12 months postpartum. Furthermore, women with ≥5 kg PPWR at 12 months postpartum had a higher GWG in the preceding pregnancy compared to women with <5 kg PPWR, except for women with an obese pre-pregnancy BMI. Women with ≥5 kg PPWR at 12 months postpartum had already a higher PPWR at 6 weeks postpartum than women who had <5 kg PPWR one year postpartum. Women in the overweight BMI category who had ≥5 kg PPWR at a year postpartum had already a higher fat percentage at six weeks postpartum. Women with normal weight and ≥5 kg PPWR at 12 months had a higher waist circumference at six weeks postpartum (Table 2).

### 3.2. Factors Associated with Evolution of Postpartum Weight and Body Composition

Pre-pregnancy BMI, amount of excessive GWG, sex of the infant, initiation of exclusive breastfeeding, a history of depressive feelings, parity, sedentary behavior, emotional eating and uncontrolled eating behaviors were significantly associated with PPWR, % GWGL, fat percentage and/or waist circumference (Table 3). No statistically significant evidence was found that restrained eating behavior, energy intake, macronutrient intake and physical activity were associated with any of the outcomes. Tables S2 and S3 show the original models with estimates, standard errors and *P*-values. Supplementary file S4 provides sample cases that illustrate the meaning of the values in Table 3.

#### 3.2.1. Pre-Pregnancy BMI

Women with overweight and obesity started off from six weeks postpartum with less PPWR (3.9 and 2.1 kg, respectively) but a higher fat percentage (36.2 and 39.3%, respectively) and higher waist circumference (87.7 and 98.2 cm, respectively) than women with normal weight (PPWR 5.5 kg; fat 30.5%; waist 81.9 cm). BMI category did not play a role in the starting point of % GWGL (66.9% in all BMI categories) (Table 3).

Between six weeks and six months postpartum, a slower monthly decrease of PPWR, fat percentage and waist circumference and a slower monthly increase in % GWGL was observed among women with overweight and obesity compared to women with normal weight. For women with obesity, there was a small monthly increase instead of a decrease in PPWR from six weeks to six months postpartum (Table 3).

**Figure 2:**
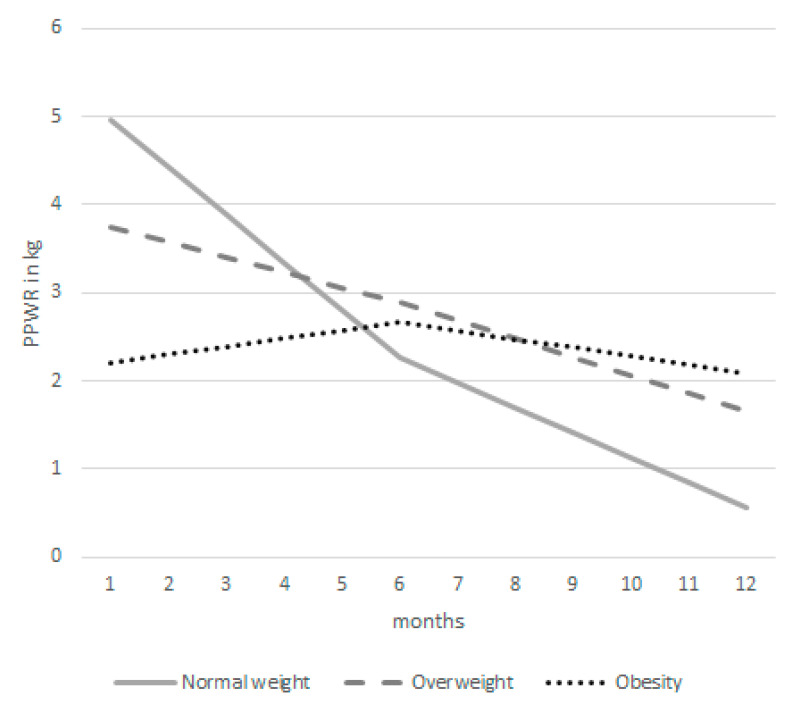
The estimated evolution of postpartum weight retention among women with normal weight, overweight and obesity based on the multivariate mixed model (N = 524 at 6 weeks, N = 395 at 6 months, N = 311 at 12 months postpartum).

Between 6 months and 12 months postpartum, the monthly evolution of all outcomes slowed down for women with normal weight. A slower evolution after six months postpartum was also seen among women with overweight for the outcomes % GWGL and waist circumference. The evolution of PPWR and fat percentage remained stable over time for women with overweight. In contrast, women with obesity showed a slightly steeper monthly decrease of PPWR and fat percentage and a slightly larger monthly increase in % GWGL from 6 months to 12 months postpartum compared to the monthly evolutions that were observed between six weeks and six months postpartum. Still, the outcomes evolved at a slower or similar pace among women with overweight and obesity compared to women with normal weight (Table 3). Figure 2 provides insight into the estimated starting point and evolution of PPWR for the three BMI groups.

#### 3.2.2. Excessive GWG

For every kilogram of GWG exceeding the guidelines, women with normal weight and obesity had one kilogram PPWR extra starting from six weeks postpartum; women with overweight had 0.8 kg of PPWR extra. The more kilograms a woman of any BMI category gained in excess of the guideline, the steeper the monthly decrease of PPWR over time (i.e., a decrease of 0.04 kg per kg excessive GWG per month) (Table 3).

Furthermore, women in all BMI groups had a 2% lower % GWGL for every kilogram exceeding the guideline and a 0.4 cm higher waist circumference starting from six weeks postpartum onwards. For fat percentage, women with normal weight had a 0.5% higher starting point for every kilogram of excessive GWG, and women with overweight and obesity, 0.2% higher. The monthly evolutions of % GWGL, fat percentage and waist circumference were not associated with the amount of kilograms exceeding the GWG guidelines for any BMI category (Table 3).

#### 3.2.3. Lifestyle

Sedentary time was a significantly associated with fat percentage starting from six weeks postpartum: every hour per day spent sitting contributed to a 0.1% higher fat percentage from six weeks postpartum onwards. A higher score in emotional eating was associated with a higher fat percentage (+0.2%) and larger waist circumference (+0.3 cm) from six weeks postpartum onwards (Table 3).

When examining determinants at six months postpartum in addition to the baseline determinants, an increasing emotional eating score between six weeks and six months postpartum was found to be associated with less favorable PPWR (+0.4 kg) and % GWGL (−0.02) from six months postpartum onwards. Likewise, an increasing score for uncontrolled eating was associated with a higher PPWR (+0.3 kg), lower % GWGL (−0.02%), and higher fat percentage (+0.3%) from six months postpartum onwards, but only for women with overweight (Table 4).

Women with overweight and obesity who started exclusive breastfeeding had a lower postpartum weight retention (−1.1 and −2.2 kg, respectively) and a higher % GWGL (+7.9% and +14.5%, respectively) starting from six weeks postpartum onwards than those who did not start exclusive breastfeeding. In addition, women with overweight who started exclusive breastfeeding had a 1.3% lower fat percentage from six weeks postpartum onwards (Table 3).

Women with obesity who had experienced depressive feelings in the past showed a 2.3 kg lower PPWR and a 17.5% higher % GWGL starting from six weeks postpartum onwards compared to women with obesity who had not experienced depressive feelings.

Physical activity, dietary intake and restrained eating behavior were not associated with PPWR, % GWGL, fat percentage or waist circumference in the models.

#### 3.2.4. Demographic Factors

Women who gave birth to a boy showed a slightly steeper monthly decrease of PPWR and waist circumference from six weeks to six months postpartum, but an opposite trend was seen from 6 months to 12 months postpartum (Table 3). At 12 months postpartum, women who gave birth to a boy were estimated to have a 0.3 kg higher PPWR compared to those who gave birth to a girl. Women who gave birth to a boy had a slightly larger monthly increase in % GWGL between six weeks and six months postpartum (+0.9%), but from 6 months to 12 months postpartum no difference was observed. In addition, a 1% higher fat percentage was observed starting from six weeks postpartum onwards among women with a boy, but no difference in the monthly evolution was found for fat percentage.

Multiparous women started at six weeks postpartum with a slightly lower % GWGL (−8%) than primiparous women, regardless of BMI category.

## 4. Discussion

This study described PPWR, % GWGL, fat percentage and waist circumference evolution in the first year after delivery among women with preceding excessive GWG and revealed how these evolutions were different according to pre-pregnancy BMI, amount of excessive GWG, and sex of the infant. In terms of lifestyle behaviors, sedentary time was associated with fat percentage, emotional eating with all outcomes, and uncontrolled eating with all outcomes except waist circumference. Additionally, initiation of exclusive breastfeeding plays an important role in PPWR and % GWGL among women with overweight and obesity and in fat percentage among women with overweight. Depressive feelings in the past contributed to PPWR and % GWGL among women with obesity and parity to % GWGL among women of all BMIs.

Pre-pregnancy BMI was found to be an important determinant of all four outcomes, stressing the importance of starting pregnancy with a healthy weight as has been previously postulated [39]. Women with overweight and obesity are estimated to have a higher PPWR at 12 months postpartum compared to women with normal weight. Excessive GWG has been previously shown to be an important risk factor for PPWR [5,6]. The current study showed that within a population of women with excessive GWG, the extent to which women exceed the GWG guidelines is a determinant of PPWR. That is, the more women gain in excess of the guideline, the more PPWR they have throughout the first year postpartum. This is likely to result in long-term PPWR and consequently an increased BMI [40]. Kilograms excessive GWG were furthermore associated with higher fat percentage and higher waist circumference, suggesting that GWG affects long-term adverse body composition and central adiposity, which is unfavorable in the light of long- term weight-related health risks.

Emotional eating and uncontrolled eating behaviors were found to be associated with the postpartum evolution of PPWR, % GWGL, and body composition. This is a novel finding, as no previous studies have investigated emotional and uncontrolled eating in the postpartum context [15]. It is remarkable that eating behaviors were important determinants rather than food intake, as previous studies suggest food intake to play a role in PPWR. However, those studies did not take eating behaviors into account [14,41].

Sedentary behavior was a determinant of fat percentage. In contrast, physical activity did not contribute to any of the models, even though physical activity was previously found to be an important determinant of (postpartum) weight and body composition [13,42]. The current study reinforces the observation that sedentary behavior is an important health determinant [43,44].

Breastfeeding has been previously suggested to affect PPWR and body composition [10,45,46]. The findings of the current study specifically support that initiation of exclusive breastfeeding is a determinant of lower PPWR and fat percentage, albeit only in women with overweight and obesity. In contrast, there was no evidence that breastfeeding duration played a role in PPWR and body composition. This would imply that starting exclusive breastfeeding, regardless of continuation, is most essential for beneficial maternal health outcomes. This is remarkable, as increased energy expenditure is generally considered to be the underlying reason for the association between breastfeeding and PPWR reduction [10,45,46]. It would be expected that duration of breastfeeding plays a role, as longer duration would imply a longer period of exposure to increased energy expenditure. However, it is also suggested that the increased energy expenditure in breastfeeding mothers is counteracted by an increased energy intake. Other unknown mechanisms could then underlie the association between breastfeeding and PPWR and body composition. The association between breastfeeding initiation and lower PPWR and fat percentage was observed in women with overweight and obesity, specifically. This emphasizes the importance of breastfeeding promotion and support in this population, especially because previous studies showed lower breastfeeding initiation among obese women compared to normal weight women [47]. Depression in general populations has been associated with both weight gain and weight loss [48]. In the perinatal context, depression was previously associated with weight gain [49]. Contrastingly, in the current study a history of depressive feelings was found to be associated with less PPWR and a greater % GWGL among women with obesity. This finding is rather atypical; however, it does emphasize that preconception (mental) health can have an impact in the long run [37,50]. This finding is especially relevant as obese women are at increased risk of depressive symptoms [51]. Future analyses of the INTER-ACT study population will explore these associations more in depth.

Whereas prenatal weight evolution is usually registered by caregivers in most countries, postpartum weight evolution is generally not systematically registered. A lack of insight into postpartum weight progression generates an inability to identify women at risk of long-term weight retention and related complications. The current study showed that women with ≥5 kg PPWR at 12 months postpartum had already a higher PPWR at six weeks postpartum compared to women with <5 kg PPWR at 12 months. This indicates that weight in early postpartum might aid identification of those at greatest risk of long-term PPWR. To this end, weighing could be integrated in the routine appointment at six weeks postpartum. This is also recommended by the American College of Obstetricians and Gynecologists [52].

The findings further suggest that postpartum lifestyle interventions should probably target eating behavior aside from food intake in women with excessive GWG. A focus on psychological support and coping strategies might be needed to improve eating behavior habits [53,54]. Women with an overweight or obese pre-pregnancy BMI need special attention as they have slower progress with PPWR than women with normal weight. In addition, initiation of exclusive breastfeeding should be further encouraged. Furthermore, both pre-pregnancy weight and GWG require optimization to prevent PPWR. This study provided evidence that limiting GWG, even if the guideline threshold of GWG is exceeded, is worthwhile.

Future research should investigate the most suitable approach to improving eating behavior and sedentary behavior among postpartum women with preceding excessive GWG. In addition, it should be investigated whether interventions targeting eating behavior and sedentary behavior aside from food intake and physical activity are effective in reducing PPWR and improving body composition in this population.

A strength of this study is the specific focus on women with previous excessive GWG and the wide range of determinants studied that potentially increase the risk of long-term PPWR within this high-risk population. Furthermore, examining body composition in addition to weight is of added value since it is linked to health outcomes.

However, this study has limitations. The high drop-out rate is a major drawback of this study, even though a reasonably large sample size was sustained. It is possible that women were, at the time of recruitment—two to three days after delivery—optimistic about participation in a postpartum study, which later might have turned out to be unrealistic for them due to the time and energy constraints common in postpartum. Furthermore, the long follow-up and extensive data collection may have placed a burden on participants. However, mixed model analyses were performed, which account for dropout. A second limitation is the relatively high level of education and predominantly white European ethnicity of the current study population. This might have compromised generalizability of the current findings to the general population. A third limitation is the use of a bio-electrical impedance analysis (BIA) device for the measurement of fat percentage. A validation study among postpartum women with overweight and obesity showed that BIA devices might underestimate fat mass when compared to dual energy X-ray absorptiometry. However, multi-frequency BIA devices as used in the current study perform better than single-frequency devices and are suitable to detect changes in fat mass over time [55], which was the purpose of the measurements in the current study. The negative consequences of using a BIA device in this study are therefore minimal.

## 5. Conclusions

Within the high-risk group of women with excessive GWG, lifestyle behaviors are associated with long-term evolution of weight and body composition. In particular eating behavior and sedentary behavior, rather than food intake and physical activity, play an important role in postpartum weight and body composition in this population. These lifestyle behaviors at six weeks postpartum continue to be associated with postpartum weight and body composition up to twelve months postpartum. Lifestyle intervention starting as early as six weeks postpartum might therefore potentially have long-term effects. Furthermore, pre-pregnancy BMI played a role in the long-term evolution of weight and body composition, emphasizing the importance of personalized health care.

## Figures and Tables

**Table 1:** Participant characteristics (N = 524).

		Sample at 6 Weeks Postpartum (Baseline) (n = 524)
Age at birth, mean (SD)		31.4 (3.9)
Parity, n (%)	Primiparous	295 (56.3)
	Multiparous	229 (43.7)
Sex of infant, n (%)	Boy	274 (52.3)
	Girl	250 (47.7)
Method of conception, n (%)	Spontaneous	473 (92)
	ART	41 (8)
	Missing	10
Education, n (%)	Up to 18 years of age	149 (29.2)
	Bachelor’s	199 (39)
	Master’s or higher	162 (31.8)
	Missing	14
Employment status, n (%)	Employed	466 (91.4)
	Unemployed	44 (8.6)
	Missing	14
Ethnicity, n (%)	White European	462 (90.6)
	Other ethnicity	48 (9.4)
	Missing	14
Pre-pregnancy BMI, n (%)	NW	256 (48.9)
	OW	187 (35.7)
	OB	81 (15.5)
Gestational weight gain in kg, median (IQR)	Among NW	19 (17–21)
	Among OW	16 (13–19)
	Among OB	14 (12–17)
PPWR in kg at 6 weeks PP, mean (SD)	Among NW	7.8 (4)
	Among OW	6.2 (4.2)
	Among OB	5.2 (5.1)
Sedentary time per day in hours, median (IQR)		5.0 (3.7–7.0)
Emotional eating score, median (IQR)		33 (11–55)
Uncontrolled eating score, mean (SD)		42 (20)
Exclusive breastfeeding at 6 weeks PP, n (%)	Yes	283 (55.8)
	No	224 (44.2)
	Missing	17
History of depressive feelings, n (%)	Yes	79 (15.8)
	No	421 (84.2)
	Missing	24

ART = assisted reproductive treatment; BMI = body mass index; NW = normal weight; OB = obesity; OW = overweight; PP = postpartum; PPWR = postpartum weight retention.

**Table 2:** Differences between participants with <5 kg and ≥5 kg postpartum weight retention at 12 months postpartum (N = 311).

		PPWR < 5 kg at 1 Year PP (n = 237)	PPWR ≥ 5 kg at 1 Year PP (n = 74)	*P*-Value *
Age at birth, mean (SD)		31.4 (3.6)	31.6 (4.3)	0.60
Parity, n (%)	Primiparous	140 (59.1)	40 (54.1)	0.45
	Multiparous	97 (40.9)	34 (45.9)	
Sex of infant, n (%)	Boy	118 (49.8)	36 (48.6)	0.86
	Girl	119 (50.2)	38 (51.4)	
Method of conception, n (%)	Spontaneous	217 (94.8)	64 (88.9)	0.08
	ART	12 (5.2)	8 (11.1)	
	Missing	8	2	
Education, n (%)	Up to 18 years of age	46 (19.7) ^a^	28 (38.4) ^a^	0.001
	Bachelor’s	92 (39.3)	30 (41.1)	
	Master’s or higher	96 (41.0) ^a^	15 (20.5) ^a^	
	Missing	3	1	
Employment status, n (%)	Employed	218 (93.2)	65 (89)	0.25
	Unemployed	16 (6.8)	8 (11)	
	Missing	3	1	
Ethnicity, n (%)	White European	216 (92.3)	67 (91.8)	0.88
	Other ethnicity	18 (7.7)	6 (8.2)	
	Missing	3	1	
Pre-pregnancy BMI, n (%)	NW	136 (57.4)	34 (45.9)	0.17
	OW	73 (30.8)	26 (35.1)	
	OB	28 (11.8)	14 (18.9)	
Gestational weight gain in kg, median (IQR)	Among NW	18 (17–20.2)	20.5 (18.4–24.3)	0.002
	Among OW	15 (13–17)	16.3 (14–19)	0.04
	Among OB	13.2 (12–15.9)	14 (11–17.6)	0.45
Exclusive breastfeeding at 6 weeks PP, n (%)	Yes	144 (65.5)	37 (56.1)	0.17
	No	76 (34.5)	29 (43.9)	
	Missing	17	8	
Exclusive breastfeeding at 6 months PP, n (%)	Yes	68 (31.3)	17 (28.3)	0.66
	No	149 (68.7)	43 (71.7)	
	Missing	20	14	
PPWR in kg at 6 weeks PP, mean (SD)	Among NW	7 (3.9)	10.5 (4.2)	<0.001
	Among OW	5.2 (3.9)	8.1 (4.3)	0.002
	Among OB	3.2 (2.8)	6.2 (4.3)	0.009
% GWGL at 6 weeks PP, mean (SD)	Among NW	64.9 (14.7)	51.8 (13.7)	<0.001
	Among OW	68.1 (21.3)	54.2 (20.7)	0.005
	Among OB	77.6 (20.2)	59.6 (28.1)	0.022
Fat % at 6 weeks PP, mean (SD)	Among NW	31.6 (4)	32.8 (3.6)	0.12
	Among OW	36.3 (2.9)	38.1 (2.9)	0.02
	Among OB	40.1 (2.2)	41.6 (4.3)	0.15
Waist circumference in cm at 6 weeks PP, median (IQR)	Among NW	81.3 (78–85.3)	84.2 (81.1–87.9)	0.02
	Among OW	88.5 (84.7–92.4)	90.6 (87.9–94.1)	0.29
	Among OB	96.5 (91.1–102.9)	102.1 (95.4–105.1)	0.09
History of depressive feelings, n (%)	Yes	23 (10)	14 (19.7)	0.03
	No	207 (90)	57 (80.3)	
	Missing	7	3	

* *P*-value for differences between groups. ^a^ Significant differences after Bonferroni correction. ART = assisted reproductive treatment; BMI = body mass index; GWGL = gestational weight gain loss; NW = normal weight; OB = obesity; OW = overweight; PP = postpartum; PPWR = postpartum weight retention.

**Table 3:** Factors at six weeks postpartum in association with evolution of weight and body composition up to 12 months postpartum (N = 524 at 6 weeks, N = 395 at 6 months, N = 311 at 12 months postpartum).

		PPWR in kg			% GWGL			Fat %			Waist Circumference in cm		
		Starting Point (W6)	Monthly Evolution W6–M6	Monthly Evolution M6–M12	Starting Point (W6)	Monthly Evolution W6–M6	Monthly Evolution M6–M12	Starting Point (W6)	Monthly Evolution W6–M6	Monthly Evolution M6–M12	Starting Point (W6)	Monthly Evolution W6–M6	Monthly Evolution M6–M12
BMI ^a^ (REF)	NW	5.5	−0.5	−0.3	66.9	3.2	2	30.5	−0.5	−0.3	81.9	−1.0	−0.5
	OW	3.9	−0.2	−0.2	66.9	2.2	2	36.2	−0.3	−0.3	87.7	−0.6	−0.4
	OB	2.1	0.1	−0.1	66.9	0.6	1.8	39.3	−0.1	−0.3	98.2	−0.5	−0.5
EGWG—per 1 kg	NW	+1.0	−0.04	−0.04	−2	NS	NS	+0.5	NS	NS	+0.4	NS	NS
	OW	+0.8	−0.04	−0.04	−2	NS	NS	+0.2	NS	NS	+0.4	NS	NS
	OB	+1.0	−0.04	−0.04	−2	NS	NS	+0.2	NS	NS	+0.4	NS	NS
Boy	NW	NS	−0.2	+0.1	NS	+0.9	NS	+1	NS	NS	NS	−0.2	+0.2
	OW	NS	−0.2	+0.1	NS	+0.9	NS	+1	NS	NS	NS	−0.2	+0.2
	OB	NS	−0.2	+0.1	NS	+0.9	NS	+1	NS	NS	NS	−0.2	+0.2
Started exclusive BF	NW	NS	NS	NS	NS	NS	NS	NS	NS	NS	NS	NS	NS
	OW	−1.1	NS	NS	+7.9	NS	NS	−1.3	NS	NS	NS	NS	NS
	OB	−2.2	NS	NS	+14.5	NS	NS	NS	NS	NS	NS	NS	NS
History of depression	NW	NS	NS	NS	NS	NS	NS	NS	NS	NS	NS	NS	NS
	OW	NS	NS	NS	NS	NS	NS	NS	NS	NS	NS	NS	NS
	OB	−2.3	NS	NS	+17.5	NS	NS	NS	NS	NS	NS	NS	NS
Multiparity	NW	NS	NS	NS	−8	NS	NS	NS	NS	NS	NS	NS	NS
	OW	NS	NS	NS	−8	NS	NS	NS	NS	NS	NS	NS	NS
	OB	NS	NS	NS	−8	NS	NS	NS	NS	NS	NS	NS	NS
Sedentary time—per 1 h daily	NW	NS	NS	NS	NS	NS	NS	+0.1	NS	NS	NS	NS	NS
	OW	NS	NS	NS	NS	NS	NS	+0.1	NS	NS	NS	NS	NS
	OB	NS	NS	NS	NS	NS	NS	+0.1	NS	NS	NS	NS	NS
Emotional eating score—per 10 points	NW	NS	NS	NS	NS	NS	NS	+0.2	NS	NS	+0.3	NS	NS
	OW	NS	NS	NS	NS	NS	NS	+0.2	NS	NS	+0.3	NS	NS
	OB	NS	NS	NS	NS	NS	NS	+0.2	NS	NS	+0.3	NS	NS
Uncontrolled eating score—per 10 points	NW	NS	NS	NS	NS	NS	NS	NS	NS	NS	NS	NS	NS
	OW	NS	NS	NS	NS	NS	NS	NS	NS	NS	NS	NS	NS
	OB	NS	NS	NS	NS	NS	NS	NS	NS	NS	NS	NS	NS

BF = breastfeeding; BMI = body mass index; EGWG = excessive kilograms gestational weight gain; M6 = 6 months postpartum; M12 = 12 months postpartum; NW = normal weight; NS = not significant; OB = obesity; OW = overweight; PPWR = postpartum weight retention; W6 = 6 weeks postpartum; % GWGL = percentage gestational weight gain loss; ^a^ Pre-pregnancy BMI

**Table 4:** Factors at six months postpartum in association with evolution of weight and body composition up to 12 months postpartum.

		PPWR in kg		% GWGL		Fat %		Waist Circumference in cm	
		Starting Point (M6)	Monthly Evolution M6–M12	Starting Point (M6)	Monthly Evolution M6–M12	Starting Point (M6)	Monthly Evolution M6–M12	Starting Point (M6)	Monthly Evolution M6–M12
Δ emotional eating score ^a^—per 10 points	NW	+0.4	NS	−0.02	NS	NS	NS	NS	NS
	OW	+0.4	NS	−0.02	NS	NS	NS	NS	NS
	OB	+0.4	NS	−0.02	NS	NS	NS	NS	NS
Δ uncontrolled eating score ^a^—per 10 points	NW	NS	NS	NS	NS	NS	NS	NS	NS
	OW	+0.3	NS	−0.02	NS	+0.3	NS	NS	NS
	OB	NS	NS	NS	NS	NS	NS	NS	NS

M6 = 6 months postpartum; M12 = 12 months postpartum; NW = normal weight; OB = obesity; OW = overweight; PPWR = postpartum weight retention; % GWGL = percentage gestational weight gain loss. ^a^ Difference in score between M6 and W6 postpartum.

## Supplementary Materials

The following are available online at https://www.mdpi.com/article/10.3390/ijerph18126344/s1, Supplementary table S1: Drop-out analyses, Table S2: multivariate mixed model of variables at 6 weeks postpartum associated with evolution of PPWR, % GWGL, fat percentage and waist circumference from 6 weeks to 12 months postpartum, Table S3: multivariate mixed model of variables at 6 weeks and 6 months postpartum associated with evolution of PPWR, % GWGL, fat percentage and waist circumference 6 weeks to 12 months postpartum, Figure S1: Evolution of PPWR in the first year postpartum for example cases A and B, Figure S2. Evolution of fat percentage in the first year postpartum for example cases A and B.

## References

[1] Endres L.K., Straub H., McKinney C., Plunkett B., Minkovitz C.S., Schetter C.D., Ramey S., Wang C., Hobel C., Raju T. (2015). Postpartum weight retention risk factors and relationship to obesity at one year. Obstet. Gynecol..

[2] Rooney B.L., Schauberger C.W., Mathiason M.A. (2005). Impact of perinatal weight change on long-term obesity and obesity-related illnesses. Obstet. Gynecol..

[3] Liu H., Zhang C., Zhang S., Wang L., Leng J., Liu D., Fang H., Li W., Yu Z., Yang X. (2014). Prepregnancy body mass index and weight change on postpartum diabetes risk among gestational diabetes women. Obesity.

[4] Seidell J.C., Halberstadt J. (2015). The global burden of obesity and the challenges of prevention. Ann. Nutr. Metab..

[5] Gallagher K., Ralph J., Petros T., Qualls C., Leeman L., Rogers R.G. (2019). Postpartum Weight Retention in Primiparous Women and Weight Outcomes in Their Offspring. J. Midwifery Women’s Health.

[6] McDowell M., Cain M.A., Brumley J. (2019). Excessive Gestational Weight Gain. J. Midwifery Women’s Health.

[7] Bogaerts A., Van den Bergh B., Nuyts E., Martens E., Witters I., Devlieger R. (2012). Socio-demographic and obstetrical correlates of pre-pregnancy body mass index and gestational weight gain. Clin. Obes..

[8] Goldstein R.F., Abell S.K., Ranasinha S., Misso M., Boyle J.A., Black M.H., Li N., Hu G., Corrado F., Rode L. (2017). Association of gestational weight gain with maternal and infant outcomes: A systematic review and meta-analysis. JAMA.

[9] Nehring I., Schmoll S., Beyerlein A., Hauner H., von Kries R. (2011). Gestational weight gain and long-term postpartum weight retention: A meta-analysis. Am. J. Clin. Nutr..

[10] Lambrinou C.P., Karaglani E., Manios Y. (2019). Breastfeeding and postpartum weight loss. Curr. Opin. Clin. Nutr. Metab. Care.

[11] McKinley M.C., Allen-Walker V., McGirr C., Rooney C., Woodside J.V. (2018). Weight loss after pregnancy: Challenges and opportunities. Nutr. Res. Rev..

[12] Nicodemus N.A. (2018). Prevention of excessive gestational weight gain and postpartum weight retention. Curr. Obes. Rep..

[13] Fadzil F., Shamsuddin K., Wan Puteh S.E., Mohd Tamil A., Ahmad S., Abdul Hayi N.S., Abdul Samad A., Ismail R., Ahmad Shauki N.I. (2018). Predictors of postpartum weight retention among urban Malaysian mothers: A prospective cohort study. Obes. Res. Clin. Pract..

[14] Siega-Riz A.M., Herring A.H., Carrier K., Evenson K.R., Dole N., Deierlein A. (2010). Sociodemographic, perinatal, behavioral, and psychosocial predictors of weight retention at 3 and 12 months postpartum. Obesity.

[15] Bijlholt M., Van Uytsel H., Ameye L., Devlieger R., Bogaerts A. (2020). Eating behaviors in relation to gestational weight gain and postpartum weight retention: A systematic review. Obes. Rev..

[16] French S.A., Epstein L.H., Jeffery R.W., Blundell J.E., Wardle J. (2012). Eating behavior dimensions. Associations with energy intake and body weight. A review. Appetite.

[17] Van Strien T., Konttinen H., Homberg J.R., Engels R.C., Winkens L.H. (2016). Emotional eating as a mediator between depression and weight gain. Appetite.

[18] Kok P., Seidell J., Meinders A. (2004). The value and limitations of the body mass index (BMI) in the assessment of the health risks of overweight and obesity. Ned. Tijdschr. Geneeskd..

[19] Ode J.J., Pivarnik J.M., Reeves M.J., Knous J.L. (2007). Body mass index as a predictor of percent fat in college athletes and nonathletes. Med. Sci. Sports Exerc..

[20] Amankwaah A.F., Hudson J.L., Kim J.E., Campbell W.W. (2019). Reductions in whole-body fat mass but not increases in lean mass predict changes in cardiometabolic health indices with exercise training among weight-stable adults. Nutr. Res..

[21] Lee D.H., Keum N., Hu F.B., Orav E.J., Rimm E.B., Willett W.C., Giovannucci E.L. (2018). Comparison of the association of predicted fat mass, body mass index, and other obesity indicators with type 2 diabetes risk: Two large prospective studies in US men and women. Eur. J. Epidemiol..

[22] Corrigan F.E., Kelli H.M., Dhindsa D.S., Heinl R.E., Al Mheid I., Hammadah M., Hayek S.S., Sher S., Eapen D.J., Martin G.S. (2017). Changes in truncal obesity and fat distribution predict arterial health. J. Clin. Lipidol..

[23] Lee J.J., Pedley A., Hoffmann U., Massaro J.M., Fox C.S. (2016). Association of changes in abdominal fat quantity and quality with incident cardiovascular disease risk factors. J. Am. Coll. Cardiol..

[24] Amato M., Guarnotta V., Giordano C. (2013). Body composition assessment for the definition of cardiometabolic risk. J. Endocrinol. Investig..

[25] Aragon A.A., Schoenfeld B.J., Wildman R., Kleiner S., VanDusseldorp T., Taylor L., Earnest C.P., Arciero P.J., Wilborn C., Kalman D.S. (2017). International society of sports nutrition position stand: Diets and body composition. J. Int. Soc. Sports Nutr..

[26] Wewege M., Van Den Berg R., Ward R., Keech A. (2017). The effects of high-intensity interval training vs. moderate-intensity continuous training on body composition in overweight and obese adults: A systematic review and meta-analysis. Obes. Rev..

[27] Lim S., O’Reilly S., Behrens H., Skinner T., Ellis I., Dunbar J. (2015). Effective strategies for weight loss in post-partum women: A systematic review and meta-analysis. Obes. Rev..

[28] Bogaerts A., Ameye L., Bijlholt M., Amuli K., Heynickx D., Devlieger R. (2017). INTER-ACT: Prevention of pregnancy complications through an e-health driven interpregnancy lifestyle intervention–study protocol of a multicentre randomised controlled trial. BMC Pregnancy Childbirth.

[29] Rasmussen K.M., Yaktine A.L., Institute of Medicine (2009). National Research Council Committee to Reexamine IOM Pregnancy Weight Guidelines. The National Academies Collection: Reports funded by National Institutes of Health. Weight Gain During Pregnancy: Reexamining the Guidelines.

[30] Guelinckx I., Devlieger R., Bogaerts A., Pauwels S., Vansant G. (2012). The effect of pre-pregnancy BMI on intention, initiation and duration of breast-feeding. Public Health Nutr..

[31] Austin M.P., Hadzi-Pavlovic D., Saint K., Parker G. (2005). Antenatal screening for the prediction of postnatal depression: Validation of a psychosocial Pregnancy Risk Questionnaire. Acta Psychiatr. Scand..

[32] Reilly N., Harris S., Loxton D., Chojenta C., Forder P., Austin M.-P. (2014). The impact of routine assessment of past or current mental health on help-seeking in the perinatal period. Women Birth.

[33] Matthys C., Meulemans A., Van der Schueren B. (2015). Development and validation of general FFQ for use in clinical practice. Ann. Nutr. Metab..

[34] Karlsson J., Persson L.-O., Sjöström L., Sullivan M. (2000). Psychometric properties and factor structure of the Three-Factor Eating Questionnaire (TFEQ) in obese men and women. Results from the Swedish Obese Subjects (SOS) study. Int. J. Obes..

[35] Anglé S., Engblom J., Eriksson T., Kautiainen S., Saha M.-T., Lindfors P., Lehtinen M., Rimpelä A. (2009). Three factor eating questionnaire-R18 as a measure of cognitive restraint, uncontrolled eating and emotional eating in a sample of young Finnish females. Int. J. Behav. Nutr. Phys. Act..

[36] Craig C.L., Marshall A.L., Sjöström M., Bauman A.E., Booth M.L., Ainsworth B.E., Pratt M., Ekelund U., Yngve A., Sallis J.F. (2003). International physical activity questionnaire: 12-country reliability and validity. Med. Sci. Sports Exerc..

[37] Bogaerts A.F., Van den Bergh B.R., Witters I., Devlieger R. (2013). Anxiety during early pregnancy predicts postpartum weight retention in obese mothers. Obesity.

[38] Althuizen E., van Poppel M.N., de Vries J.H., Seidell J.C., van Mechelen W. (2011). Postpartum behaviour as predictor of weight change from before pregnancy to one year postpartum. BMC Public Health.

[39] Poston L., Caleyachetty R., Cnattingius S., Corvalán C., Uauy R., Herring S., Gillman M.W. (2016). Preconceptional and maternal obesity: Epidemiology and health consequences. Lancet Diabetes Endocrinol..

[40] Catalano P.M., Shankar K. (2017). Obesity and pregnancy: Mechanisms of short term and long term adverse consequences for mother and child. BMJ.

[41] Dalrymple K.V., Flynn A.C., Relph S.A., O’Keeffe M., Poston L. (2018). Lifestyle Interventions in Overweight and Obese Pregnant or Postpartum Women for Postpartum Weight Management: A Systematic Review of the Literature. Nutrients.

[42] Keller C., Ainsworth B., Records K., Todd M., Belyea M., Vega-López S., Permana P., Coonrod D., Nagle-Williams A. (2014). A comparison of a social support physical activity intervention in weight management among post-partum Latinas. BMC Public Health.

[43] Fazzi C., Saunders D.H., Linton K., Norman J.E., Reynolds R.M. (2017). Sedentary behaviours during pregnancy: A systematic review. Int. J. Behav. Nutr. Phys. Act..

[44] Nayak M., Peinhaupt M., Heinemann A., Eekhoff M.E., van Mechelen W., Desoye G., van Poppel M.N. (2016). Sedentary behavior in obese pregnant women is associated with inflammatory markers and lipid profile but not with glucose metabolism. Cytokine.

[45] Lima N.P., Bassani D.G., Silva B., Motta J.V.S., Magalhães E.I.S., Barros F.C., Horta B.L. (2019). Association of breastfeeding, maternal anthropometry and body composition in women at 30 years of age. Cad. Saude Publica.

[46] Martin J.E., Hure A.J., Macdonald-Wicks L., Smith R., Collins C.E. (2014). Predictors of post-partum weight retention in a prospective longitudinal study. Matern. Child. Nutr..

[47] Turcksin R., Bel S., Galjaard S., Devlieger R. (2014). Maternal obesity and breastfeeding intention, initiation, intensity and duration: A systematic review. Matern. Child. Nutr..

[48] Privitera G.J., Misenheimer M.L., Doraiswamy P.M. (2013). From weight loss to weight gain: Appetite changes in major depressive disorder as a mirror into brain-environment interactions. Front. Psychol..

[49] Slomian J., Honvo G., Emonts P., Reginster J.Y., Bruyère O. (2019). Consequences of maternal postpartum depression: A systematic review of maternal and infant outcomes. Women’s Health.

[50] Lang A.Y., Boyle J.A., Fitzgerald G.L., Teede H., Mazza D., Moran L.J., Harrison C. (2018). Optimizing preconception health in women of reproductive age. Minerva Ginecol..

[51] Molyneaux E., Poston L., Ashurst-Williams S., Howard L.M. (2014). Pre-pregnancy obesity and mental disorders during pregnancy and postpartum: A systematic review and meta-analysis. Pregnancy Hypertens. Int. J. Women’s Cardiovasc. Health.

[52] The American College of Obstetricians and Gynecologists (2018). Committee opinion no. 736: Optimizing postpartum care. Obstet. Gynecol..

[53] Paans N.P.G., Bot M., Brouwer I.A., Visser M., Gili M., Roca M., Hegerl U., Kohls E., Owens M., Watkins E. (2020). Effects of food-related behavioral activation therapy on eating styles, diet quality and body weight change: Results from the MooDFOOD Randomized Clinical Trial. J. Psychosom. Res..

[54] Paans N.P.G., Bot M., Brouwer I.A., Visser M., Roca M., Kohls E., Watkins E., Penninx B. (2018). The association between depression and eating styles in four European countries: The MooDFOOD prevention study. J. Psychosom. Res..

[55] Ellegård L., Bertz F., Winkvist A., Bosaeus I., Brekke H. (2016). Body composition in overweight and obese women postpartum: Bioimpedance methods validated by dual energy X-ray absorptiometry and doubly labeled water. Eur. J. Clin. Nutr..

