# Supplementary material for: Evolution of Postpartum Weight and Body Composition after Excessive Gestational Weight Gain: The Role of Lifestyle Behaviors—Data from the INTER-ACT Control Group
Source: Int J Environ Res Public Health. 2021 Jun 11;18(12):6344. doi: 10.3390/ijerph18126344 (PMC8296169; doi:10.3390/ijerph18126344)
Supplement: Supplementary file 1 [file ijerph-18-06344-s001.zip › ijerph-1132558-supplementary.pdf]

Supplementary tables

Supplementary Table S1: Drop-out analyses

		**Dropped out before 12 months postpartum (N=213)**	**Followed up until 12 months postpartum (N=311)**	**P-value***
Age at birth, mean (SD)		31.5 (4.1)	31.4 (3.7)	0.94
Parity, n (%)	Primiparous	115 (54)	180 (57.9)	0.38
	Multiparous	98 (46)	131 (42.1)	
Sex of infant, n (%)	Boy	120 (56.3)	154 (49.5)	0.12
	Girl	93 (43.7)	157 (50.5)	
Method of conception, n (%)	Spontaneous	192 (90.1)	281 (93.4)	0.19
	ART	21 (9.9)	20 (6.6)	
	Missing	0	10	
Education, n (%)	Up to 18 years of age	75 (36.9) [a]	74 (24.1) [a]	0.003
	Bachelor	77 (37.9)	122 (39.7)	
	Master or higher	51 (25.1) [a]	111 (36.2) [a]	
	Missing	10	4	
Employment status, n (%)	Employed	183 (90.1)	283 (92.2)	0.43
	Unemployed	20 (9.9)	24 (7.8)	
	Missing	10	4	
Ethnicity, n (%)	White European	179 (88.2)	283 (92.2)	0.13
	Other ethnicity	24 (11.8)	24 (7.8)	
	Missing	10	4	
Pre-pregnancy BMI, n (%)	NW	86 (40.4) [a]	170 (54.7) [a]	0.006
	OW	88 (41.3)	99 (31.8)	
	OB	39 (18.3)	42 (13.5)	
Gestational weight gain in kg, median (IQR)	Among NW	19.2 (17-21.1)	19 (17-21)	0.70
	Among OW	17 (14-20)	15 (13-17.1)	0.007
	Among OB	14.5 (12-17)	13.7 (11.9-16)	0.48
Exclusive breastfeeding at 6 weeks PP, n (%)	Yes	100 (49.8)	183 (59.8)	0.03
	No	101 (50.2)	123 (40.2)	
	Missing	12	5	
PPWR in kg at 6 weeks PP, mean (SD)	Among NW	7.8 (3.6)	7.7 (4.1)	0.83
	Among OW	6.5 (4.3)	5.9 (4.2)	0.37
	Among OB	6.3 (6.2)	4.2 (3.7)	0.07
History of depressive feelings, n (%)	Yes	42 (21.1)	37 (12.3)	0.009
	No	157 (78.9)	264 (87.7)	
	Missing	14	10	

* P-value for differences between drop-outs and followed-up sample at 12 months postpartum.

[a] Significant differences after Bonferroni correction.

ART = assisted reproductive treatment; BMI = body mass index; NW = normal weight; OB = obesity; OW = overweight; PP = postpartum; PPWR = postpartum weight retention.

Supplementary Table S2: multivariate mixed model of variables at 6 weeks postpartum associated with evolution of PPWR, %GWGL, fat percentage and waist circumference from 6 weeks to 12 months postpartum.

	PPWR in kg (N=492)			%GWGL (N=492)			Fat % (N=471)			Waist circumference in cm (N=493)		
	**B**	**SE**	**P-value**	**B**	**SE**	**P-value**	**B**	**SE**	**P-value**	**B**	**SE**	**P-value**
**Intercept**	5.5055	0.3465	<0.001	66.9	1.757	<0.001	30.5038	0.4973	<0.001	81.9283	0.5843	<0.001
**Months**	-0.5395	0.06641	<0.001	3.245	0.3532	<0.001	-0.5135	0.04591	<0.001	-1.0063	0.07514	<0.001
**Monthsminus6*M6**	0.2549	0.08724	0.004	-1.246	0.5103	0.02	0.2096	0.06575	0.002	0.4854	0.1116	<0.001
**Overweight**	-1.5927	0.6882	0.02				5.7105	0.6909	<0.001	5.7585	0.6759	<0.001
**Obesity**	-3.3951	0.9355	<0.001				8.7648	0.8441	<0.001	16.2521	0.9354	<0.001
**Months*overweight**	0.3699	0.08533	<0.001	-1.091	0.4646	0.02	0.2607	0.07212	<0.001	0.3920	0.1008	<0.001
**Months*obesity**	0.6332	0.1190	<0.001	-2.673	0.6698	<0.001	0.4377	0.1045	<0.001	0.4848	0.1409	<0.001
**Monthsminus6*M6*overweight**	-0.2922	0.1214	0.02	1.247	0.6895	0.07	-0.1889	0.1063	0.08	-0.3088	0.1502	0.04
**Monthsminus6*M6*obesity**	-0.4462	0.1673	0.008	2.511	0.9651	0.01	-0.2914	0.1500	0.05	-0.3830	0.2092	0.07
**Boy**							-1.0437	0.3002	<0.001			
**Months*boy**	-0.1517	0.06048	0.01	0.9461	0.3656	0.01				-0.1742	0.08004	0.03
**Monthsminus6*M6*boy**	0.2483	0.09726	0.01	-1.381	0.5756	0.02				0.3839	0.1273	0.003
**ExcessiveGWG**	0.9814	0.06306	<0.001	-2.03	0.2338	<0.001	0.4507	0.07829	<0.001	0.3875	0.07811	<0.001
**Months*excessiveGWG**	-0.03755	0.007668	<0.001									
**Overweight*excessiveGWG**	-0.1914	0.08769	0.03				-0.2641	0.1023	0.01			
**Obesity*excessiveGWG**							-0.2495	0.1267	0.05			
**Obesity*depression**	-2.3371	1.0229	0.02	17.53	5.163	0.001						
**Overweight*breastfeeding**	-1.1175	0.5378	0.04	7.916	2.163	<0.001	-1.3133	0.5326	0.01			
**Obesity*breastfeeding**	-2.1614	0.8893	0.02	14.48	3.07	<0.001						
**Multiparity**				-8.04	1.603	<0.001						
**Sedentarytime**							0.000319	0.000127	0.01			
**Emotionaleating**							0.01883	0.005310	<0.001	0.03112	0.009026	<0.001

GWG = gestational weight gain; M6 = 6 months postpartum; PPWR = postpartum weight retention; %GWGL = percentage gestational weight gain loss

Supplementary Table S3: multivariate mixed model of variables at 6 weeks and 6 months postpartum associated with evolution of PPWR, %GWGL, fat percentage and waist circumference 6 weeks to 12 months postpartum.

	PPWR in kg (N=353)			%GWGL (N=353)			Fat % (N=354)			Waist circumference in cm (N=369)		
	**B**	**SE**	**P-value**	**B**	**SE**	**P-value**	**B**	**SE**	**P-value**	**B**	**SE**	**P-value**
**Intercept**	5.3419	0.4043	<0.001	0.6728	0.02019	<0.001	30.4290	0.5562	<0.001	81.8438	0.6298	<0.001
**Months**	-0.5410	0.07004	<0.001	0.03236	0.003742	<0.001	-0.5016	0.04697	<0.001	-0.9889	0.07635	<0.001
**Monthsminus6*M6**	0.2681	0.09090	0.004	-0.01311	0.005285	0.01	0.1945	0.06682	0.004	0.4618	0.1122	<0.001
**Overweight**	-1.5280	0.8357	0.07				5.7218	0.8173	<0.001	6.1251	0.7500	<0.001
**Obesity**	-3.7140	1.2495	0.003				8.8583	0.9801	<0.001	14.6878	1.0267	<0.001
**Months*overweight**	0.3681	0.09018	<0.001	-0.00972	0.004957	0.05	0.2510	0.07506	0.001	0.3677	0.1044	<0.001
**Months*obesity**	0.6621	0.1249	<0.001	-0.02951	0.007083	<0.001	0.4760	0.1076	<0.001	0.5416	0.1434	<0.001
**Monthsminus6*M6*overweight**	-0.3176	0.1256	0.01	0.01315	0.007127	0.07	-0.1898	0.1090	0.08	-0.3058	0.1530	0.05
**Monthsminus6*M6*obesity**	-0.4858	0.1723	0.005	0.02838	0.009935	0.005	-0.3413	0.1520	0.03	-0.4386	0.2095	0.04
**Boy**							-1.1399	0.3469	0.001			
**Months*boy**	-0.1577	0.06483	0.02	0.01067	0.003926	0.007				-0.1805	0.08168	0.03
**Monthsminus6*M6*boy**	0.2440	0.1019	0.02	-0.01377	0.005964	0.02				0.3946	0.1289	0.002
**ExcessiveGWG**	1.0109	0.07688	<0.001	-0.01916	0.002934	<0.001	0.4210	0.08914	<0.001	0.3377	0.08858	<0.001
**Months*excessiveGWG**	-0.03956	0.008187	<0.001									
**Overweight*excessiveGWG**	-0.3071	0.1085	0.005				-0.2196	0.1199	0.07			
**Obesity*excessiveGWG**							-0.3153	0.1632	0.05			
**Obesity*depression**	-3.1860	1.3035	0.02	0.2192	0.06251	<0.001						
**Overweight*breastfeeding**	-0.6095	0.6858	0.38	0.06743	0.02575	0.009	-1.0562	0.6685	0.12			
**Obesity*breastfeeding**	-1.7540	1.2027	0.15	0.1474	0.03693	<0.001						

**Multiparity**				-0.08697	0.01928	<0.001						
**Sedentarytime**							0.000332	0.000141	0.02			
**Emotionaleating**							0.01628	0.006149	0.009	0.03297	0.009924	0.001
**M6*Δemotionaleating[1]**	0.03927	0.01300	0.003	-0.00241	0.000803	0.003						
**M6*overweight*Δuncontrolledeating[1]**	0.03227	0.01564	0.04	-0.00239	0.000966	0.01	0.02928	0.01045	0.006	0.02730	0.01455	0.06

GWG = gestational weight gain; M6 = 6 months postpartum; PPWR = postpartum weight retention; %GWGL = percentage gestational weight gain loss

[1] Change between 6 weeks and 6 months postpartum

Supplementary file S4: Example cases to illustrate table 3

To illustrate table 3, two fictive postpartum women are compared, 'A' and 'B'. Both women A and B had a normal weight pre-pregnancy BMI, gave birth to a girl (reference), started exclusive breastfeeding, had no history of depression (reference), and were primiparous (reference). However, woman A and woman B differ in terms of kilograms of excessive weight gain in pregnancy, sedentary time and emotional eating score. Woman A gained 2 kg's in excess of the GWG guidelines, reported sitting 4 hours per day, and had an emotional eating score of 11. Woman B gained 6 kg's in excess of the GWG guidelines, reported sitting 8 hours per day, and had an emotional eating score of 56.
The evolution of PPWR for woman A is calculated as follows:

- Starting point at six weeks postpartum:
  5.5 kg [for normal pre-pregnancy BMI] + 1 kg * 2 [for 2 kg EGWG] = 7.5 kg
- Monthly evolution from six weeks to six months postpartum:
  -0.5 kg [for normal pre-pregnancy BMI] -0.04 kg * 2 [for 2 kg EGWG] = -0.58 kg per month
- Monthly evolution from six months to twelve months postpartum:
  -0.3 kg [for normal pre-pregnancy BMI] -0.04 kg * 2 [for 2 kg EGWG] = -0.38 kg per month

The evolution of PPWR for woman B would be calculated as follows:

- Starting point at six weeks postpartum:
  5.5 kg [for normal pre-pregnancy BMI] + 1 kg * 6 [for 6 kg EGWG] = 11.5 kg
- Monthly evolution from six weeks to six months postpartum:
  -0.5 kg [for normal pre-pregnancy BMI] -0.04 kg * 6 [for 6 kg EGWG] = -0.74 kg per month
- Monthly evolution from six months to twelve months postpartum:
  -0.3 kg [for normal pre-pregnancy BMI] -0.04 kg * 6 [for kg EGWG] = -0.54 kg per month

Figure S1 displays the expected PPWR of woman A and woman B based on these calculations.

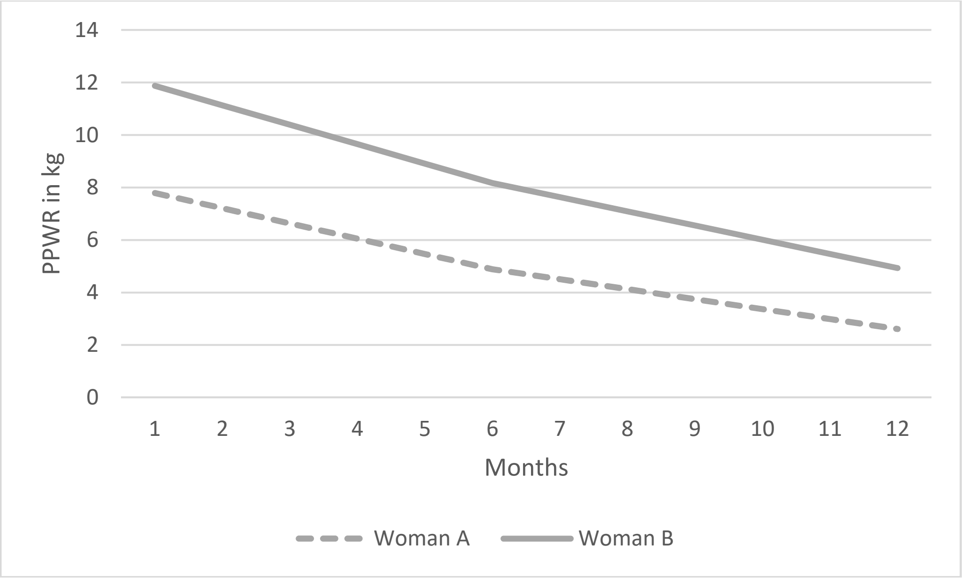

Figure S1. Evolution of PPWR in the first year postpartum for example cases A and B

Sedentary time, emotional eating score and starting exclusive breastfeeding did not significantly contribute to the model for PPWR and did thus not play a role in this calculation. However, sedentary time and emotional eating score did contribute to the model for fat percentage.

The evolution of fat percentage for woman A is calculated as follows:

- Starting point at six weeks postpartum:
  30.5 [for normal pre-pregnancy BMI] + 0.5 * 2 [for 2 kg EGWG] + 0.1 * 4 [for 4 sedentary hours per day] + 0.2 * 1.1 [for an emotional eating score of 11] = 32.12 %
- Monthly evolution from six weeks to six months postpartum:
  -0.5 % [for normal pre-pregnancy BMI]
- Monthly evolution from six months to twelve months postpartum:
  -0.3 % [for normal pre-pregnancy BMI]

The evolution of fat percentage for woman B is calculated as follows:

- Starting point at six weeks postpartum:
  30.5 [for normal pre-pregnancy BMI] + 0.5 * 6 [for 6 kg EGWG] + 0.1 * 8 [for 8 sedentary hours per day] + 0.2 * 5.6 [for an emotional eating score of 56] = 35.42 %
- Monthly evolution from six weeks to six months postpartum:
  -0.5 % [for normal pre-pregnancy BMI]
- Monthly evolution from six months to twelve months postpartum:
  -0.3 % [for normal pre-pregnancy BMI]

Besides pre-pregnancy BMI, none of the other variables significantly contributed to the slope of the monthly evolution in the model for fat percentage. Figure S2 displays the expected evolution of fat percentage of woman A and woman B based on these calculations.

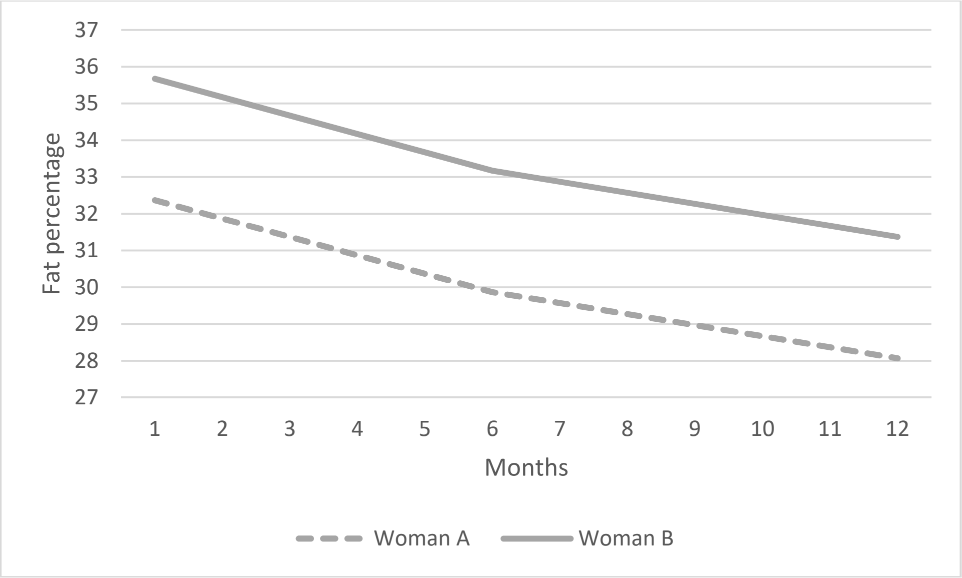

Figure S2. Evolution of fat percentage in the first year postpartum for example cases A and B
